# Prognostic and predictive value of radiomics signatures in stage I‐III colon cancer

**DOI:** 10.1002/ctm2.31

**Published:** 2020-04-30

**Authors:** Weixing Dai, Shaobo Mo, Lingyu Han, Wenqiang Xiang, Menglei Li, Renjie Wang, Tong Tong, Guoxiang Cai

**Affiliations:** ^1^ Department of Colorectal Surgery Fudan University Shanghai Cancer Center Shanghai China; ^2^ Department of Oncology Shanghai Medical College Fudan University Shanghai China; ^3^ Department of Radiology Fudan University Shanghai Cancer Center Shanghai China

**Keywords:** colon cancer, overall survival, radiomics, relapse free survival

## Abstract

Accurate identification of patients with poor prognosis after radical surgery is essential for clinical management of colon cancer. Thus, we aimed to develop death and relapse specific radiomics signatures to individually estimate overall survival (OS) and relapse free survival (RFS) of colon cancer patients. In this study, 701 stage I‐III colon cancer patients were identified from Fudan University Shanghai Cancer Center. A total of 647 three‐dimensional features were extracted from computed tomography images. LASSO Cox was used to identify the significantly death‐ and relapse‐associated features and to build death and relapse specific radiomics signatures, respectively. A total of 13 death‐specific and 26 relapse‐specific features were identified from 647 screened radiomics features. The developed signatures can divide patients into two groups with significantly different death (Hazard Ratio (HR): 3.053; 95% CI, 1.78‐5.23; *P* < .001) or relapse risk (HR: 2.794; 95% CI, 1.87‐4.16; *P* < .001). Time‐dependent Relative operating characteristic curve showed that the signatures performed better than any other clinicopathological factors in predicting OS (AUC: 0.768; 95% CI, 0.745‐0.791) and RFS (AUC: 0.744; 95% CI, 0.687‐0.801). Further, survival decision curve analyses confirmed the good clinical utility of the two radiomics signatures. In conclusion, we successfully developed death‐ and relapse‐specific radiomics signatures that can accurately predict OS and RFS, which may facilitate personalized treatment.

Colorectal cancer (CRC) is currently one of the most common malignant diseases, and once patients were diagnosed with metastatic colon cancer, their prognosis would be extremely poor.^1^ Therefore, it is urgently needed to identify more predictive factors to help identify those patients who may benefit from more aggressive treatment.

To date, American Joint Commission on Cancer (AJCC) tumor‐node‐metastasis (TNM) staging system has been established as the most important risk factor for colon cancer.^2^ However, because of the great tumor heterogeneity, it is common to see that the prognosis of patients with the same tumor stage varied distinctively. Though many other clinical and pathological features have been identified to help to predict the survival of colon cancer patients, the prognostic accuracy of these factors was still insufficient.^3^, ^4^, ^5^ Consequently, more accurate prognostic factors or predictive model should be developed.

Noninvasive computed tomography (CT) has been established as a routine examination for preoperative assessment in colon cancer. Recently, CT‐based radiomics is an emerging novel methodology that can extract high‐dimensional imaging features for in‐depth analysis and for facilitating treatment decision‐making.^6^, ^7^, ^8^ In contrast to traditional imaging features, radiomics was supposed to be able to reveal innate disease characteristics and even genetic features.^7^ In a high‐throughput manner, many kinds of multi‐feature signatures have been developed based on radiomics to improve tumor detection, tumor classification, therapeutic response evaluation, and prognosis prediction in solid tumors.^9^, ^10^, ^11^, ^12^ In predicting prognosis, the radiomics signatures have been reported to be a reliable prognostic model in breast and gastric cancer.^13^, ^14^ However, to our best knowledge, no previous studies have been conducted to develop a signature based on radiomics to predict prognosis for colon cancer.

The aim of this retrospective study was to develop two specific radiomics signatures to individually predict long‐term overall survival (OS) and relapse free survival (RFS) in colon cancer patients; such two predictive models would be helpful in improving risk stratification and therapeutic strategy.

Colon cancer patients diagnosed from April 2012 and December,2015 in Fudan University Shanghai Cancer Center (FUSCC) were identified in this study. Patients who were pathologically confirmed with stage I‐III colon cancer and received radical primary resection were retrospective identified. Patients without contrast‐enhanced abdominal pelvic CT and follow‐up information were filtered. Finally, a total of 701 eligible stage I‐III patients were identified this study. Preoperative blood samples were obtained from every hospitalized patient by routine to examine the status of CEA and CA19_9. Postoperative pathological features will be read and classified by experienced pathologist. Immunohistochemistry will be further performed to determine the mismatch repair status. This follow‐up was censored on 10 October 2019 with a median follow‐up time of 55 months. The study program was approved by the FUSCC Ethical Committee and Institutional Review Board and written informed consent was obtained from all patients. The detailed information of image acquisition, segmentation, radiomics feature extraction, reproducibility evaluation, and signature construction was described in the Supporting Information.

Among the 701 colon cancer patients identified from FUSCC database, 367 (52.4%) patients were over 60 years old. A total of 391 (55.8%) were male and 310 (44.2%) were male patients. Three hundred fifty (49.9%) patients were diagnosed with left‐sided colon cancer and 351 (50.1%) with left‐sided colon cancer. Eighty‐one (11.6%) patients were stage I, 335 (47.8%) were stage II, and 285 (40.7%) were stage III colon cancer. Distribution of colon cancer in grade I, grade II, and III was 21 (3.0%), 625 (89.2%), and 55 (7.8%), respectively. The more detailed baseline information was showed in Table S1. Figure S1 showed the LASSO coefficient profiles of the death (A) and relapse (B) associated features. A coefficient profile plot was produced against the log (λ) sequence. Under 10‐fold cross‐validation, 13 death specific features and 26 relapse specific features were identified (Table S2).

The distribution of death/relapse‐specific risk scores and the corresponding events status were shown in Figure 1A (left panel) and Figure 1B (left panel), suggesting a positive correlation between risk score and death or relapse rate. To show the prognostic accuracy of death‐ and relapse‐specific radiomics signatures, time‐dependent relative operating characteristic (ROC) at 1, 3, and 5 years were performed (Figures 1A and 1B, middle panel). Based on median value of death risk score (0.121), patients were separated into low‐risk group (N = 350) and high‐risk group (N = 351). The 5‐year OS for patients in low death risk group was 93%, compared with 77.1% in high death risk group (Hazard Ratio (HR): 3.053; 95% CI, 1.78‐5.23; *P* < .001; Figure 1A, right panel). Similar result was found between low and high relapse risk group. The 5‐year RFS for low‐risk patients was significantly shorter than cases in high relapse risk group (HR: 2.794; 95% CI, 1.87‐4.16; *P* < .001; Figure 1B, right panel) (median relapse risk score: 0.189). The relationship between radiomics signatures and clinicopathological features was further analyzed and it showed that both death and recurrence high‐risk groups determined by radiomics signatures were significantly associated with advanced tumor stage (Table S3).

**FIGURE 1:**
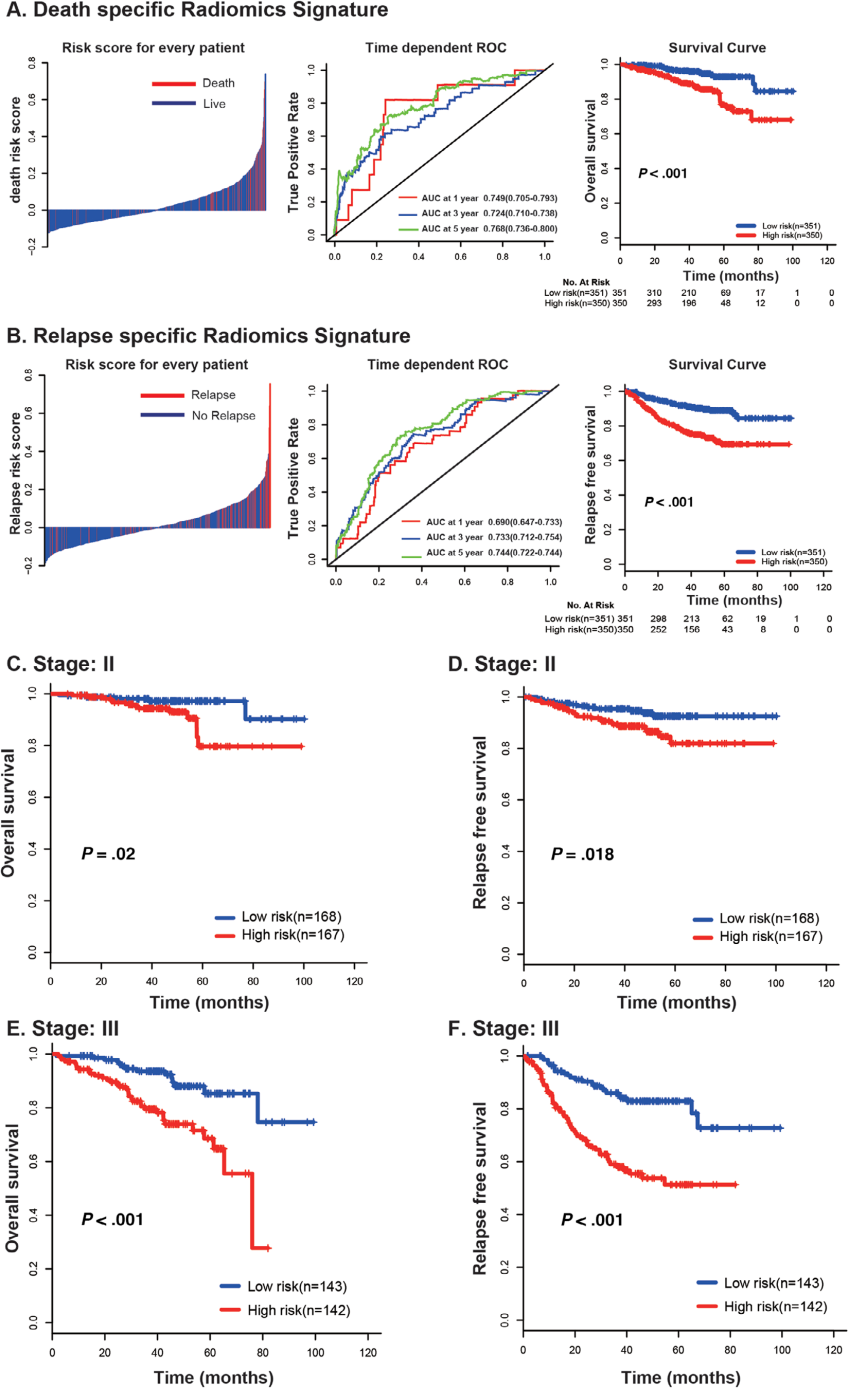
Distribution of radiomics risk score, time‐dependent relative operating characteristiccurves at 1, 3, and 5 years, and Kaplan‐Meier survival curves of patients at low and high risk of death (**A**) and relapse (**B**). Stratified Kaplan‐Meier survival analysis of death and relapse signatures based on tumor stage. (**C**) Death signature in stage II; (**D**) relapse signature in stage II; (**E**) death signature in stage III; (**F**) relapse signature in stage III

To analyze the independent prognostic role of radiomics signatures, multivariate analysis was further conducted and it showed that the death‐ and relapse‐specific radiomics signatures remained their robustness and independent role in predicting OS (Table S4) and RFS (Table S5) after adjusting clinical factors. Stratified analysis based on AJCC TNM stage showed that the risk classification based on two signatures performed well in both stage II and III colon cancer patients (Figure 1C‐F). Accurate prediction of prognosis in colon cancer is critically important for clinical practice. Based on these two radiomics signatures, we can divide patients into two groups with significantly death or relapse group. Furthermore, the developed two signatures showed independent prognostic value in predicting OS and RFS, demonstrating the incremental value of the radiomics sign to the current staging or risk evaluation system of colon cancer.

To test whether the developed death‐ and relapse‐specific radiomics signatures performed more accurately than other prognostic factors, time‐dependent ROC was conducted and the result showed that death (AUC: 0.768; 95% CI, 0.745‐0.791) and relapse (AUC: 0.744; 95% CI, 0.687‐0.801) specific radiomics signatures had significantly higher predictive accuracy than any other clinicopathological features in predicting OS (Figure 2A) and RFS (Figure 2B).

**FIGURE 2:**
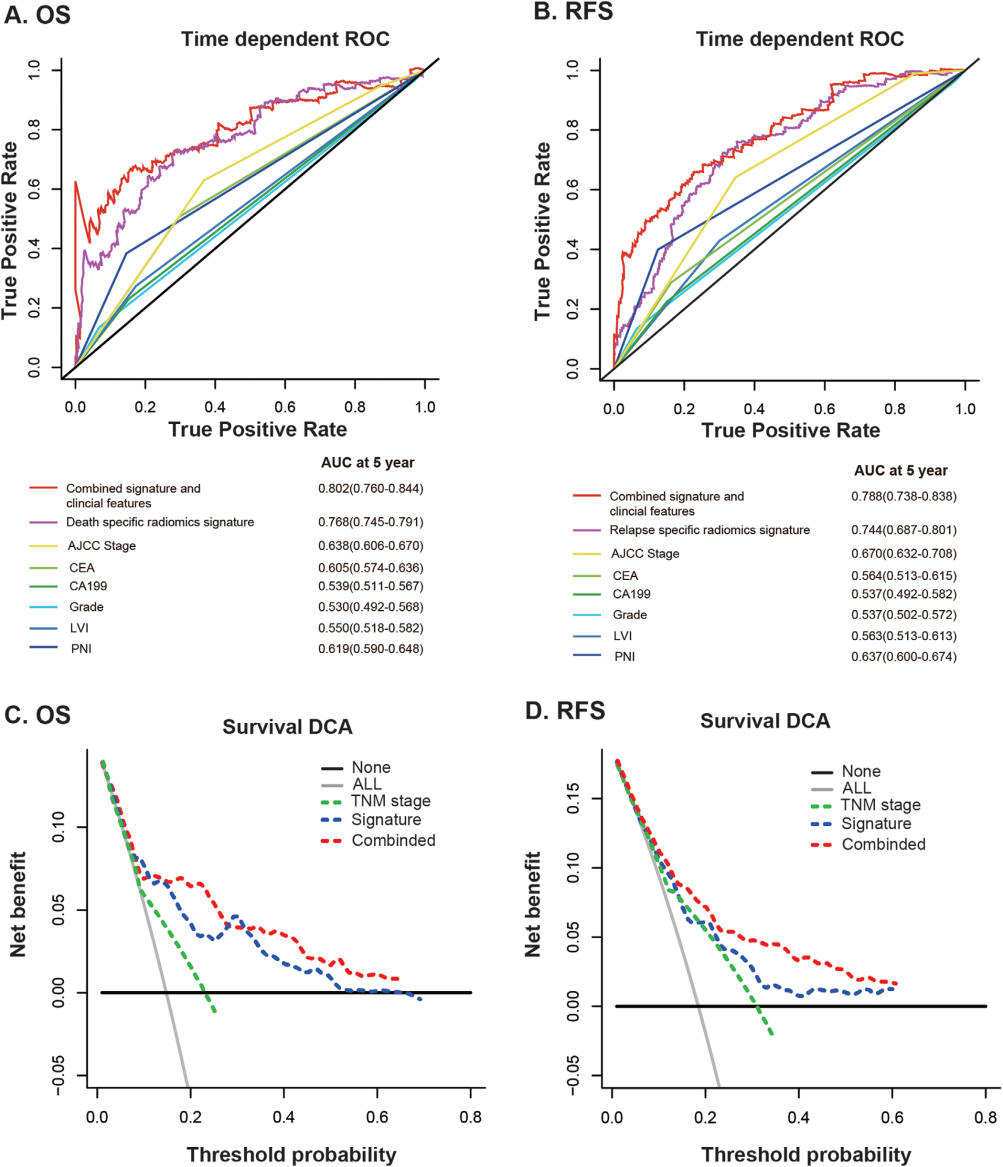
Time‐dependent relative operating characteristic curves at 5 years compare the prognostic accuracy in predicting OS (**A**) and RFS (**B**) of radiomics signatures with clinicopathological features including American Joint Commission on Cancer (AJCC) tumor stage, grade, CEA status, CA19_9 status, Lymphatic vascular invasion (LVI), and Peripheral nervous invasion (PNI). Decision curve analysis at 5 years of OS (**C**) and RFS (**D**) for the radiomics signature, tumor stage, and the two combined model. The y‐axis measures the net benefit.

Clinical utility is an another important factor for evaluating the value of prognostic models. The decision curve analysis for OS and RFS prediction at 5‐year for AJCC tumor stage, radiomics signatures, and the integrated model was illustrated in Figure 2. The decision curves revealed that both the two radiomics signatures brought significantly more benefit than AJCC tumor stage, indicating that the radiomics signature outperformed clinical features with more accuracy. The decision curve analysis (DCA) curves suggested that if the threshold probability is over 5%, radiomics signatures will add more net benefit than the “treat all” or “treat none” strategy. In addition, with the combination of radiomics signatures and clinical prognostic factors, the integrated model achieved notably more clinical utility.

Unlike invasive tissue biopsy, imaging can assess the characteristics of tumor noninvasively. However, the shortage of traditional imaging is obviously. For the imaging interpretation is subjective or qualitative, the clinical significance has long been debated. Recently, the radiomics is attracting attention from the medical field. The imaging acquisition and analysis based on radiomics allowed high‐throughput extraction of informative imaging features and even can quantify the characteristic of a tissue, which are impossible for human clinical experience. The advances in radiomics have renewed our mind on medical imaging and a large amount of studies have revealed the great potential of radiomics to be used as predictive tool for prognosis assessment.^15^, ^16^

Taking a step forward, several researchers have developed radiomics signatures for the prediction of lymph node metastasis^11^ and the neoadjuvant chemoradiotherapy response evaluation in CRC.^17^ In our previous work, we even analyzed the relationship between features and genetic mutations based on radiomics and developed a signature to predict the existence of gene mutation.^9^ In addition, the radiomics was also used to predict the prognosis for patients with breast, gastric, and lung cancer.^18^ To date, the application of radiomics in colon cancer for OS and RFS prediction has not been reported. In this present study, both morphological features and internal texture features were quantified when we performed the radiomics process. A total of 13 death‐specific and 26 relapse‐specific features were identified from 647 screened radiomics features by using the LASSO regression method. Indeed, previous studies confirmed that combined analysis of a panel consisting of multiple features as a signature was a much better method compared to the individual factors analysis in predicting prognosis, and the radiomics signature has been suggested as the most promising management approach.^19^ Therefore, the radiomics‐based risk score was calculated by combing the multi‐radiomics features, and it was successfully confirmed that radiomics signature had a good performance in prediction OS and RFS.

In this present study, several limitations cannot be avoided. First, as a retrospective analysis, there are possible selection bias in our study. Second, although the prognostic ability and generalization of the three‐dimensional feature of primary tumor were assessed in this study, the performance of features from regional lymph nodes warrants further investigation. Third, although good performance of the developed signatures has been demonstrated, no external validation sets were used to substantiate their prognostic and predictive value. Therefore, further validation of patients from other cancer centers should be performed.

Our study showed the developed CT‐based radiomics signatures have the potential to be used as a tool for death and relapse risk stratification for colon cancer. Integration of radiomics signature and clinical features will further facilitate the prediction of postoperative survival and help to individualize treatment and follow‐up scheme for colon cancer patients.

## AUTHOR CONTRIBUTIONS

TT and GC conceptualized and designed the study. WD, SM, LH, and ML performed analysis. WX and RW interpreted the data. WD and SM drafted the manuscript. TT and GC revised the manuscript. All authors read and approved the final manuscript

## CONFLICT OF INTEREST

The authors declare no conflict of interest.

## Supporting information

SUPPORTING INFORMATION

## Data Availability

All the data are available on reasonable request.
