# Supplementary material for: Prognostic and predictive value of radiomics signatures in stage I‐III colon cancer
Source: Clin Transl Med. 2020 Apr 30;10(1):288–93. doi: 10.1002/ctm2.31 (PMC7240849; doi:10.1002/ctm2.31)
Supplement: Supplementary file 1 — SUPPORTING INFORMATION [file CTM2-10-288-s001.docx]

**Supporting Information**

***Image acquisition, segmentation, radiomics feature extraction and reproducibility evaluation***

All selected patients underwent contrast-enhanced abdominal CT at our institution with 64-row spiral CT scanners (Philips Healthcare, Siemens Healthcare) using a current of 200 mA and a tube voltage of 120 kV. All CT images were reconstructed with the standard reconstruction kernel, including 5.0 mm slice thickness, 5.0 mm increment, 1.4 or 0.9 pitch, a 512×512 matrix and 4.11 cm field of view. The CT digital imaging and communication in medicine (DICOM) images were retrieved from the picture archiving and communication system (PACS). Regions of interests (ROIs) of both the primary lesion was semi-automatically segmented along the lesion contour in the largest cross-sectional area by an operator using ITK-SNAP software (v 3.6.0; www.itksnap.org) and then validated by a senior radiologist (T.T.) with 20-year work experience. Contouring was drawn within the borders of the primary tumor masses to avoid partial volume effects, including adjacent air, fat, vessels, normal tissue, and surrounding organs.

To ensure reproducibility, we repeated the generation of radiomics features twice within an interval of at least one month following the same procedure. Intra-class correlation coefficients (ICCs) were used to assess the intra- and inter-observer agreement. Like our previous report, an ICC of 0.81-1.00 was set as almost perfect agreement, 0.61-0.80 as substantial agreement, 0.41-0.60 as moderate, 0.21-0.40 as fair agreement, and 0-0.20 as no agreement[1]. In this study, satisfactory inter- and intra-observer reproducibility was achieved with ICC > 0.6 between the features form same radiologist at baseline and at least 1 month later.

As described in previous work, we have explored a feature-based approach to extract and quantify meaningful and reliable information from images[2, 3]. All 647 3D features were extracted based on the original image and its corresponding filtered image, including the features from the categories of histogram, shape, gray-level co-occurrence matrix and gray-level run-length matrix. After feature extraction, z-score normalization was performed.

***Signature Construction and performance evaluation***

By using LASSO Cox regression model to analyze the relationship between radiomics features and OS or RFS, we identified a list of death specific features and relapse specific features. A standard formula of death or relapse risk radiomics-score for each patient was calculated combining the value of the features and LASSO Cox regression coefficients. Based on the median value of death and relapse risk score, patients were divided into two groups with high and low risk, respectively.

Radiomics risk score=∑(feature values×coefficients)

To investigate the predictive accuracy of the developed the radiomics signature, time-dependent receiver operating characteristic (ROC) analysis was used. Survival decision curve analysis (DCA) was further used to evaluate the net benefit derived from the two radiomics signatures. The clinical utility could be demonstrated by quantifying the net benefits of a series of threshold probabilities in the queue. Decision curve analysis examined the theoretical relationship between the threshold death or relapse probability at 5 year of colon cancer and the relative value of false-positive and false-negative results to determine the predictive ability of the prediction model[4].

1. Collins GS, Reitsma JB, Altman DG, and Moons KGM, Transparent Reporting of a Multivariable Prediction Model for Individual Prognosis or Diagnosis (TRIPOD): The TRIPOD Statement*.* Eur Urol, 2015. 67(6): 1142-1151.

2. Yang L, Dong D, Fang M, Zhu Y, Zang Y, Liu Z, Zhang H, Ying J, Zhao X, and Tian J, Can CT-based radiomics signature predict KRAS/NRAS/BRAF mutations in colorectal cancer? Eur Radiol, 2018. 28(5): 2058-2067.

3. Shen C, Liu Z, Guan M, Song J, Lian Y, Wang S, Tang Z, Dong D, Kong L, Wang M, Shi D, and Tian J, 2D and 3D CT Radiomics Features Prognostic Performance Comparison in Non-Small Cell Lung Cancer*.* Transl Oncol, 2017. 10(6): 886-894.

4. Vickers AJ and Cronin AM, Traditional statistical methods for evaluating prediction models are uninformative as to clinical value: towards a decision analytic framework*.* Semin Oncol, 2010. 37(1): 31-8.

| Table 1. Baseline clinicopathological features of the patients from FUSCC database. | | |
| --- | --- | --- |
|  | N | % |
| Age |  |  |
| <60 | 334 | 47.6 |
| ≥60 | 367 | 52.4 |
| Sex |  |  |
| Male | 391 | 55.8 |
| Female | 310 | 44.2 |
| MMR status |  |  |
| pMMR | 559 | 79.7 |
| dMMR | 142 | 20.3 |
| Tumor Location |  |  |
| Left side | 350 | 49.9 |
| Right side | 351 | 50.1 |
| Stage |  |  |
| I | 81 | 11.6 |
| II | 335 | 47.8 |
| III | 285 | 40.7 |
| Grade |  |  |
| I | 21 | 3 |
| II | 625 | 89.2 |
| III | 55 | 7.8 |
| CEA status |  |  |
| Low | 472 | 67.3 |
| High | 229 | 32.7 |
| CA19_9 status |  |  |
| Low | 585 | 83.5 |
| High | 116 | 16.5 |
| LVI |  |  |
| Negative | 569 | 81.2 |
| Positive | 132 | 18.8 |
| PNI |  |  |
| Negative | 574 | 81.9 |
| Positive | 127 | 18.1 |

LVI: Lymph-Vascular invasion; PNI: peripheral invasion

| Table 2. LASSO Cox coefficients of death and relapse specific radiomics features. |  |  |  |
| --- | --- | --- | --- |
| **Death specific features** |  | |  |
| name | Coefficients | |  |
| N25_ori_glszm_GLNU | 0.149 | |  |
| N25_ori_fos_skewness | 0.153 | |  |
| N25_ori_fos_mean | -0.406 | |  |
| N25_ori_fos_krutosis | -0.533 | |  |
| N25_Coif7_glszm_SZSE | 0.461 | |  |
| N25_Coif6_glszm_SZSE | -0.123 | |  |
| N25_Coif6_glcm_entropy | 0.395 | |  |
| N25_Coif5_glszm_GLV | -0.645 | |  |
| N25_Coif5_fos_skewness | -0.464 | |  |
| N25_Coif1_glszm_SZLGE | -1.039 | |  |
| N25_Coif1_glcm_inverse_variance | 0.529 | |  |
| N25_Coif1_fos_range | 0.603 | |  |
| N25_Coif1_fos_minimum | -0.504 | |  |
| **Death specific features** |  | |  |
| name | Coefficients | |  |
| N25_Coif7_glszm_ZSV | -0.076 | |  |
| N25_Coif6_glcm_covariance | -0.004 | |  |
| N25_Coif6_glcm_correlation | -0.248 | |  |
| N25_Coif5_glcm_covariance | -0.215 | |  |
| N25_Coif5_glcm_cluster_shade | -0.225 | |  |
| N25_Coif5_fos_skewness | -0.017 | |  |
| N25_Coif5_fos_root_mean_square | -0.313 | |  |
| N25_Coif5_fos_median | 0.032 | |  |
| N25_Coif5_fos_mean | 0.456 | |  |
| N25_Coif4_glrlm_SRLGLE | 0.422 | |  |
| N25_Coif3_glcm_cluster_shade | -0.108 | |  |
| N25_Coif3_fos_median | 0.14 | |  |
| N25_Coif3_fos_mean | -0.375 | |  |
| N25_Coif2_glrlm_SRLGLE | -0.694 | |  |
| N25_Coif2_glrlm_LGLRE | 0.33 | |  |
| N25_Coif1_glszm_ZSP | -0.389 | |  |
| N25_Coif1_glszm_SZSE | -0.092 | |  |
| N25_Coif1_glszm_SZLGE | -0.453 | |  |
| N25_Coif1_glszm_LZSE | 0.071 | |  |
| N25_Coif1_glszm_LZLGE | -0.487 | |  |
| N25_Coif1_glszm_LGLZE | 0.038 | |  |
| N25_Coif1_glszm_HGLZE | -0.091 | |  |
| N25_Coif1_glcm_covariance | -0.296 | |  |
| N25_Coif1_fos_skewness | 0.133 | |  |
| N25_Coif1_fos_mean_absulute_deviation | -0.462 | |  |
| N25_Coif1_fos_krutosis | -0.765 | |  |
|  |  |  | |

Table 3. Correlation analysis between radiomics signatures and clinical factors.

|  | | OS signature | | P | RFS signature | | P |
| --- | --- | --- | --- | --- | --- | --- | --- |
|  |  | low risk | high risk |  | low risk | high risk |  |
| Age | <60 | 171 | 163 | 0.522 | 170 | 164 | 0.624 |
|  | ≥60 | 179 | 188 |  | 180 | 187 |  |
| sex | Male | 169 | 222 | <0.001 | 187 | 204 | 0.211 |
|  | Female | 181 | 129 |  | 163 | 147 |  |
| MMR | pMMR | 276 | 283 | 0.560 | 276 | 283 | 0.560 |
|  | dMMR | 74 | 68 |  | 74 | 68 |  |
| Stage | I | 51 | 30 | 0.044 | 57 | 24 | <0.001 |
|  | II | 162 | 173 |  | 163 | 172 |  |
|  | III | 137 | 148 |  | 130 | 155 |  |
| Location | Left side | 163 | 187 | 0.076 | 181 | 169 | 0.345 |
|  | Right side | 187 | 164 |  | 169 | 182 |  |
| Grade | I | 11 | 10 | 0.968 | 16 | 5 | 0.049 |
|  | II | 312 | 313 |  | 306 | 319 |  |
|  | III | 27 | 28 |  | 28 | 27 |  |
| CA19_9 | Low | 298 | 287 | 0.229 | 301 | 284 | 0.070 |
|  | High | 52 | 64 |  | 49 | 67 |  |
| CEA | Low | 246 | 226 | 0.096 | 255 | 217 | 0.002 |
|  | High | 104 | 125 |  | 95 | 134 |  |
| LVI | Negative | 286 | 283 | 0.713 | 289 | 280 | 0.342 |
|  | Positive | 64 | 68 |  | 61 | 71 |  |
| PNI | Negative | 286 | 288 | 0.908 | 292 | 282 | 0.289 |
|  | Positive | 64 | 63 |  | 58 | 69 |  |

LVI: Lymph-Vascular invasion; PNI: peripheral invasion

| Table 4. Univariate and Multivariate Cox regression analysis of OS. | | | | | |  |  |
| --- | --- | --- | --- | --- | --- | --- | --- |
|  |  | Univariate |  |  |  | Multivariate | |
|  | HR | 95%CI | P |  | HR | 95%CI | P |
| Age |  |  | <0.001 |  |  |  | 0.002 |
| <60 | 1 |  |  |  | 1 |  |  |
| ≥60 | 2.395 | 1.42-4.04 |  |  | 2.392 | 1.39-4.11 |  |
| Sex |  |  | 0.563 |  |  |  |  |
| Male | 1 |  |  |  |  |  |  |
| Female | 0.867 | 0.53-1.41 |  |  |  |  |  |
| MMR status |  |  | 0.868 |  |  |  |  |
| pMMR | 1 |  |  |  |  |  |  |
| dMMR | 1.054 | 0.59-1.87 |  |  |  |  |  |
| Tumor Location |  |  |  |  |  |  |  |
| Left side | 1 |  | 0.451 |  |  |  |  |
| Right side | 1.202 | 0.74-1.94 |  |  |  |  |  |
| Stage |  |  | <0.001 |  |  |  | <0.001 |
| I | 1 |  |  |  | 1 |  |  |
| II | 2.53 | 0.58-10.91 |  |  | 1.807 | 0.41-7.93 |  |
| III | 8.779 | 2.13-36.16 |  |  | 5.322 | 1.23-22.97 |  |
| Grade |  |  | 0.003 |  |  |  | 0.31 |
| I | 1 |  |  |  | 1 |  |  |
| II | 2.039 | 0.28-14.75 |  |  | 1.237 | 0.16-9.09 |  |
| III | 5.894 | 0.76-45.01 |  |  | 2.064 | 0.25-16.55 |  |
| CEA status |  |  | 0.018 |  |  |  | 0.986 |
| Low | 1 |  |  |  | 1 |  |  |
| High | 1.817 | 0.12-2.94 |  |  | 1.005 | 0.59-1.69 |  |
| CA19_9 status |  |  | 0.006 |  |  |  | 0.09 |
| Low | 1 |  |  |  | 1 |  |  |
| High | 2.144 | 1.24-3.68 |  |  | 1.679 | 0.92-3.06 |  |
| LVI |  |  | 0.005 |  |  |  | 0.697 |
| Negative | 1 |  |  |  | 1 |  |  |
| Positive | 2.103 | 1.24-3.54 |  |  | 1.127 | 0.61-2.06 |  |
| PNI |  |  | <0.001 |  |  |  | 0.095 |
| Negative | 1 |  |  |  | 1 |  |  |
| Positive | 3.185 | 1.94-5.22 |  |  | 1.631 | 0.91-2.89 |  |
| Death radiomics signature | 170.777 | 49.4-590.28 | <0.001 |  | 99.099 | 24.48-401.98 | <0.001 |

LVI: Lymph-Vascular invasion; PNI: peripheral invasion

| Table 5. Univariate and Multivariate Cox regression analysis of RFS. | | | | | |  |  |
| --- | --- | --- | --- | --- | --- | --- | --- |
|  |  | Univariate |  |  |  | Multivariate | |
|  | HR | 95%CI | P |  | HR | 95%CI | P |
| Age |  |  | 0.201 |  |  |  | 0.002 |
| <60 | 1 |  |  |  | 1 |  |  |
| ≥60 | 0.79 | 0.54-1.14 |  |  | 2.392 | 1.39-4.11 |  |
| Sex |  |  | 0.751 |  |  |  |  |
| Male | 1 |  |  |  |  |  |  |
| Female | 1.06 | 0.74-1.53 |  |  |  |  |  |
| MMR status |  |  | 0.133 |  |  |  |  |
| pMMR | 1 |  |  |  |  |  |  |
| dMMR | 0.68 | 0.41-1.12 |  |  |  |  |  |
| Tumor Location |  |  | 0.411 |  |  |  |  |
| Left side | 1 |  |  |  |  |  |  |
| Right side | 1.165 | 0.81-1.68 |  |  |  |  |  |
| Stage |  |  | <0.001 |  |  |  | <0.001 |
| I | 1 |  |  |  | 1 |  |  |
| II | 3.114 | 0.95-10.14 |  |  | 2.12 | 0.65-6.99 |  |
| III | 9.739 | 3.07-30.86 |  |  | 5.13 | 1.59-17.09 |  |
| Grade |  |  | 0.004 |  |  |  | 0.118 |
| I | 1 |  |  |  | 1 |  |  |
| II | 1.917 | 0.47-7.77 |  |  | 1.072 | 0.26-4.39 |  |
| III | 4.398 | 1.02-19.05 |  |  | 1.881 | 0,42-8.33 |  |
| CEA status |  |  | <0.001 |  |  |  | 0.224 |
| Low | 1 |  |  |  | 1 |  |  |
| High | 1.822 | 1.26-2.63 |  |  | 1.269 | 0.86-1.86 |  |
| CA19_9 status |  |  | 0.007 |  |  |  | 0.324 |
| Low | 1 |  |  |  | 1 |  |  |
| High | 1.8 | 1.18-2.75 |  |  | 1.258 | 0.79-1,98 |  |
| LVI |  |  | <0.001 |  |  |  | 0.697 |
| Negative | 1 |  |  |  | 1 |  |  |
| Positive | 2.125 | 1.43-3.16 |  |  | 1.008 | 0.65-1.58 |  |
| PNI |  |  | <0.001 |  |  |  | <0.001 |
| Negative | 1 |  |  |  | 1 |  |  |
| Positive | 3.67 | 2.53-5.325 |  |  | 2.444 | 1.64-3.65 |  |
| Relapse radiomics signature | 93.376 | 29.53-295.19 | <0.001 |  | 59.65 | 19.67-180.88 | <0.001 |

LVI: Lymph-Vascular invasion; PNI: peripheral invasion


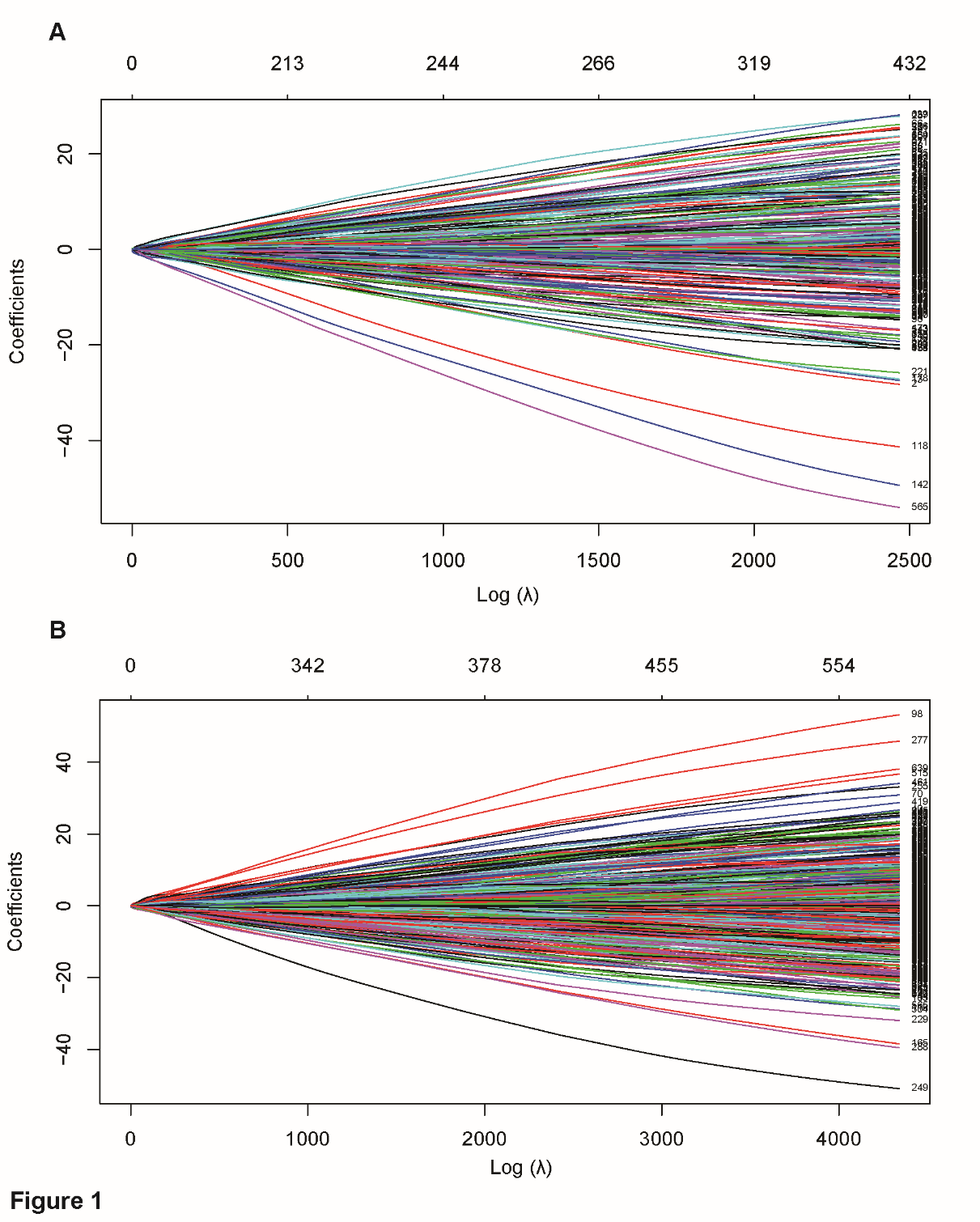
**Supplementary Figure 1.** LASSO coefficient profiles of the 647 features in predicting OS (A) and RFS (B). A vertical line is drawn at the value chosen by 10-fold cross-validation.
